# Efficacy and safety of a dual boosted protease inhibitor-based regimen, atazanavir and fosamprenavir/ritonavir, against HIV: experience in a pediatric population

**DOI:** 10.1186/1471-2334-12-179

**Published:** 2012-08-06

**Authors:** Stefano Rusconi, Vania Giacomet, Chiara Mameli, Alessandra Viganò, Ottavia Viganò, Fulvio Adorni, Massimo Galli, Gian Vincenzo Zuccotti

**Affiliations:** 1 Department of Biomedical and Clinical Science “Luigi Sacco”, Infectious Diseases, University of Milan, Milan, Italy; 2 Department of Biomedical and Clinical Science “Luigi Sacco”, Pediatrics, University of Milan, Milan, Italy; 3 ITB-CNR, Segrate, Milan, Italy; 4 Sezione di Malattie Infettive, Dipartimento di Scienze Biomediche e Cliniche (DIBIC) "Luigi Sacco", Universita' degli Studi di Milano, via G.B. Grassi 74, Milano, 20157, Italy

## Abstract

**Background:**

Although dual-boosted protease inhibitors regimen is not recommended in children with HIV infection, such a strategy could be useful in subjects with a complex resistance profile. This study was aimed at assessing the long term efficacy and safety of a double-boosted protease inhibitor combination, fosamprenavir (fAVP) and atazanavir/ritonavir (ATV/r) in a cohort of HIV-infected children and adolescents who had failed with nucleoside reverse transcriptase inhibitors.

**Methods:**

Seven vertically infected children and adolescents who had previously failed highly active antiretroviral therapy and were resistant to nucleoside reverse transcriptase inhibitors, received a dual protease inhibitor (PI) regimen including fAVP plus ATV/r for 42 months. The patients were assessed at baseline, every month for the first 24 weeks of therapy and every 3 months until month 32. Physical examination, CD4+ cell count, HIV-RNA viral load, lipid profile and hepatic function were assessed throughout the follow up.

**Results:**

During the study no serious adverse events were reported. CD4 absolute number increased over-time in all subjects. At baseline the median HIV-RNA was 6562 cp/mL (ranging 1048 -102772 cp/mL) and rapidly decreased below the limit of detection (50 cp/mL) after 2 months of the new treatment and remained undetectable in all cases through the entire study period. At the beginning of the study all cases showed a normal lipid profile. During the study period, 4/7 subjects showed total cholesterol, low density lipoprotein and triglyceride levels >97^th^ cent.le for the males and 94^th^ cent.le for the females. HDL cholesterol showed protective values. Hepatic enzymes remained stable during the entire observation, whereas total bilirubin showed toxicity II/III grade in 6/7 subjects. No change in fat redistribution and insulin resistance was observed.

**Conclusion:**

Dual-boosted protease inhibitor therapy was virologically and immunologically effective and it could be considered as a possible alternative to a rescue regimen in children and adolescents. However, hypercholesterolemia and hypertriglyceridemia need close follow-up and may limit the use of this therapeutic option.

## Background

The introduction of potent combination antiretroviral (ARV) therapy has been found to improve disease progression and prolonged survival in HIV-infected adults and children [1-3]. Despite the clinical favourable outcome observed with ARV, it is difficult to choose an effective alternative regimen after multiple therapeutic failures in many patients. Approximately 40 to 50% of adult patients receiving ARV regimen containing a protease inhibitor (PI) experience virologic failure [4,5], mainly due to side effects and unfavourable pill burden. Moreover the long term control of viral replication is even lower in paediatric patients [6]. Most patients show primary mutations to protease inhibitors (PIs), resistance to non-nucleoside reverse transcriptase inhibitors (NNRTI) and low activity of nucleoside reverse transcriptase inhibitors (N[n]RTIs). In this setting, the idea of using a dual-boosted PI regimen with low-dose boosting ritonavir may be considered.

The rationale of using a dual-boosted PI regimen is to reach synergistic or additive activity against HIV and to further increase the genetic barrier to resistance, to obtain high plasma concentrations of both PIs with only one boost and to avoid toxicity with N(n)RTI-sparing regimens [7].

The short-term efficacy of double-boosted PI in naïve adults has been disappointing [8,9], while studies in treatment experienced patients showed more favourable results. In an observational cohort study of 128 heavily pre-treated patients, the combination of lopinavir/ritonavir (LPV/r) and saquinavir (SQV) obtained a virological response (HIV-RNA < 400 copies/ml) at week 48 [10,1]. In a recent randomized study in patients who had failed NNRTI-based regimen, comparing single-boosted PI with double- boosted PI, Siripasson et al found an acceptable virological outcome without differences in the two groups [11].

In pediatric patients, dual boosted PI regimen is not recommended because few data are available in this population [12]. However, two recent studies showed that dual-boosted SQV and LPV may be an effective and safe alternative for a second-line regimen in pre-treated children, nevertheless a close follow up is necessary because dislipidemia can occur during treatment [13,14]. Therefore, we decided to test this therapeutic option in a long term observational cohort of 7 HIV-infected children.

## Methods

Our observational study evaluated the immuno-virologic efficacy and the safety profile of dual boosted PI-based therapy in a cohort of vertically HIV- infected pediatric patients who had failed nucleoside N(n)RTI based therapy. Inclusion criteria were: ongoing viral replication, multiple virological failures using a N(n)RTI-containing regimen, and presence of resistance-associated mutations to N(n)RTI and NNRTI in the current genotype.

Genotype resistance testing (Siemens Healthcare Diagnostics Trugene® HIV-1, Siemens Healthcare Diagnostics**,** 1717 Deerfield Road, Deerfield, IL 60015-0778, USA) was performed in each patient before the introduction of the dual boosted PI-based regimen. Mutations were defined according to International AIDS Society-USA Drug Resistance Mutation Group [15]. The interpretation of resistance mutations was done through the Stanford HIV Db, Genotypic Resistance Interpretation Algorithm, version 6.2.0.

Each patient switched to a dual PI-based regimen consisting of fosamprenavir (fAVP) and atazanavir (ATV) boosted ritonavir (RTV) in association with lamivudine (3TC) and this regimen was maintained over 42 months. Lamivudine was included in the drug regimen due to its low viral fitness maintenance in the presence of M184V mutation. The administration schedule was fAPV 18 mg/kg bid, ATV 150 mg bid with the booster of RTV 100 mg bid and 3TC 4 mg/kg bid. Adherence was assessed through the subject’s pill count and a self-reported evaluation.

CD4 cell counts, HIV-RNA viral load, serum chemistry, fasting lipid levels and clinical assessments were performed in each patient at baseline (T0), 3 months after the switch and every six months until month 42. HIV viral load was assessed by Branched-DNA, Chiron Diagnostics, Emeryville, CA, USA with a lower limit of detection < 50 copies/mL; CD4 cell count was performed using standard flow cytometry. Serum chemistry and lipid levels were assessed by standard laboratory methods.

The efficacy endpoint was the achievement of plasma HIV-RNA < 50 copies cp/mL with stable or increasing CD4 cell counts through 42 months. Virological failure was defined as having 2 consecutive HIV-RNA > 50 cp/mL both during the study period and at the end of the clinical observation (42 months).

Safety assessments included lipid profiles defined as changes in total cholesterol (TC), high-density lipoprotein cholesterol (HDL), low-density lipoprotein cholesterol (LDL) and triglycerides (TG) as well as hepatic toxicity: aspartate aminotransferase (AST), alanine aminotransferase (ALT), total bilirubin (TB), gamma glutamyl transferase (γGT). Lipid levels were defined according to the reference by age and gender in adolescents [16,17].

Lipodistrophy was annually assessed using a checklist of standard clinical signs for fat redistribution completed by the pediatrician for each child [18]. Children were considered to have fat redistribution if they had one or more of major clinical signs. A combined subtype of fat redistribution was defined as showing at least one sign of peripheral lipoatrophy and at least one of central lipohypertrophy.

Homeostatic Model Assessment (HOMA) index was evaluated in this cohort by an oral glucose tolerance test (OGTT) during the annual follow up visit to check for insulin resistance. The HOMA index was calculated as the product of the fasting plasma insulin level (μU/mL) and the fasting plasma glucose level (mmol/L), divided by 22.5. HOMA index cut-offs, from the 5^th^ to the 95^th^ percentile.

Categorical variables are given as the number or percentage of subject with the characteristic of interest. Continuous variables are reported as median, lower (25^th^ percentile), and upper quartile (75^th^ percentile). The comparison between the baseline and 42 month time-point was conducted with the Wilcoxon non-parametric test. The level of statistical significance was set at P <0.05. SPSS 15 for Windows was the statistical software package used for the analyses (SPSS, Chicago, IL, USA).

A formal approval from the Ethical Committee was not required. Our institution’s IRB does not require approval for therapeutic interventions which are part of the regular clinical practice. The children were started on this treatment as part of their standard care prescribed by their physician and no treatment was given for the purposes of this study.

## Results

The cases, 5 males and 2 females, showed the following CDC stage: A1 (1 patient), A2 (1), A3 (1), B3 (2), C2 (1).The median age of the patients was 15.96 years and the interquartile range was 10.82 to 18.93 years. One patient was co-infected with Hepatitis C virus, but his condition was clinically stable and no laboratory evidence of decreased hepatic function was evident. All patients presented the inclusion criteria as previously specified. Prior to the introduction of the new regimen, 5/7 patients were taking a N(n)RTI-based antiretroviral regimen and 2/7 an antiretroviral regimen including 2 N(n)RTIs and 1 PI.

**Figure 1:**
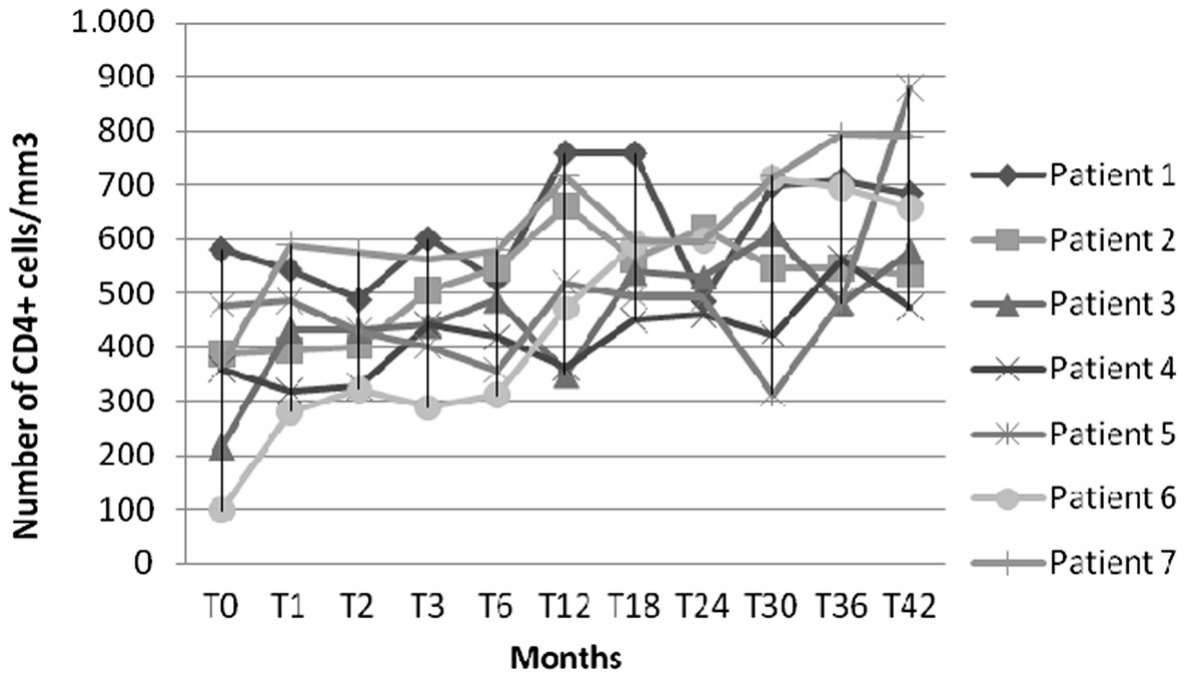
CD4 cell count profiles in HIV-infected children and adolescents treated with double-boosted protease inhibitors during the study period.

The double boosted-PI regimen plus 3TC achieved an excellent virological and immunological response**.** As shown in Figure 1, CD4 absolute number increased over time in all subjects. At baseline, the median and the interquartile range for the CD4 absolute number and the percentage were 364 (217-478)/μL and 18 (16.9-21.2) %, respectively, whereas at month 42 the values were 618 (534-684)/μL and 26.6 (21.4-28.3) %, respectively. This increase was statistically significant (p < 0.05).

At baseline the median and HIV-RNA was 6562 cp/ml, ranging from 1048 to 102772 cp/ mL. Viral load showed a rapid decrease below the detection limit (50 cp/ mL) after 2 months of the new treatment in all subjects and remained undetectable in all cases throughout of study period.

The results of the genotype resistance tests are shown in Table 1. As shown, all pediatric subjects but 1 exhibited resistance to the majority of N(n)RTI and this was the main reason that promted us to use a dual boosted PI-based regimen. The only child without resistance mutations received a dual boosted PI-based regimen due to hematologic and metabolic toxicities.

**Table 1:** Genotypic resistance mutations before switching to a double-boosted protease inhibitors regimen

	**NNRTI**					**PI**	**Resistant Drugs**
**Patient**	**98 (G)**	**103 (R/N)**	**108 (I)**	**135 (L/I/V)**	**283 (I)**		
P1	G			L		63 P, 71 V, 77 wt/I, 93 L	NVP L-lR
P2		N	I	N	I	10 V, 35 D	EFV and NVP L-lR
P3					wt/I	77I	none
P4						63 P, 93 L	none
P5		V		V		35 D, 60 E, 63 P	none
P6						No mutations	none
P7		R		T		10 V, 13 wt/V, 63 P, 77 I	none

**Figure 2:**
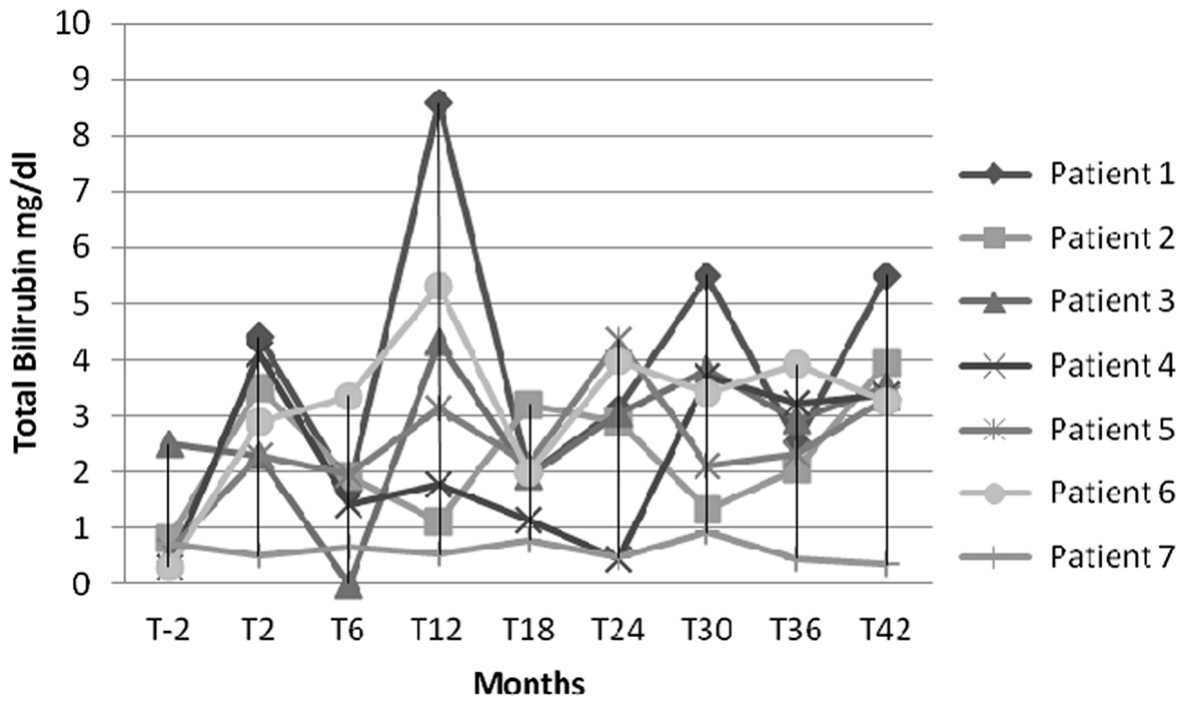
Total bilirubin profile in HIV-infected children and adolescents treated with double-boosted protease inhibitors during the study period.

Liver enzymes showed a certain degree of variation, but did not cause any treatment interruption during the study period. At baseline, the median and range for ALT were 40 (18-78) U/L, whereas they were 33 (30-40) U/L at month 42. AST resulted 37 (22-70) U/L and 32 (29-38) U/L and γGT was 27 (18-42) U/L and 28 (18-34) U/L, at baseline and month 42, respectively. All hepatic enzymes remained quite stable during the entire observation time without any statistical difference. As shown in Figure 2, TB varied more extensively at different time intervals. Overall one subject maintained a normal level, whereas the other 4 subjects showed a grade 2. One patient showed a grade 3 toxicity at month 12, while a single patient reached the grade 4 of toxicity at month 12, 30 and 42 [17]. At baseline, the TB median and range values were 0.65 (0.30-0.80) mg/dL and reached 3.37 (3.27-3.96) mg/dL at month 42; this result was statistically significant (P = 0.046).

**Figure 3:**
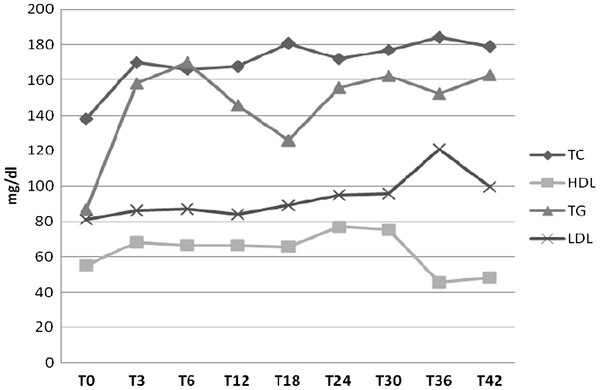
Mean values of lipids (TC, HDL, TG, LDL) in HIV-infected children and adolescents treated with double-boosted protease inhibitors during the study period.

Fasting lipid profiles (TC, HDL, LDL, and TG) are shown in Figure 3. TC showed a significant increase (p < 0.05) from baseline to month 42, 143 (112-157) mg/dL and 194 (160-203) mg/dL, respectively. TC values > 97^th^ cent.le for males and 94 ^th^cent.le for females were detected in 4/7 patients (57%). TG increased from baseline (78 mg/dL [59-106]) to month 42 (175 mg/dL [135-194]) with a significant difference (P < 0.05). TG values >97^th^ percentile were detected in 4/7 patients. (57 %) LDL cholesterol remained stable over-time, with baseline values of 75 (64-105) mg/dl and 106 (92-116) at month 42. LDL values > 97^th^ cent.le for males and >95^th^ cent.le for females were detected in patients 4/7 patients (57%). HDL exhibited protective values during the follow up.

At baseline, one out of 7 patients had lipohypertrophy consisting in truncal fat accumulation, “buffalo hump” and breast enlargement. In this single patient, lipohypertrophy remained stable during the follow up. No other patients developed signs of lipodistrophy during the double-boosted PI therapy.

At baseline all patients had a normal HOMA index (3.8 [1.2-10.1)], which was maintained stable during the follow up.

## Discussion

This study showed that at month 42 a combination regimen of fAVP + ATV/r obtained an excellent virological (100% of subjects with HIV-RNA <50 copies/ml) and a good immunological (9% median CD4 increase) outcome in HIV-infected children and adolescents although there was an association of a worsening lipid profile. Our results showed a similar efficacy compared to the other two available studies of double-boosted PI in experienced HIV-infected children. These two studies assessed the efficacy of the combination of SQV and LPV/r in a cohort of 50 HIV-infected children; the first and the second assessment were performed at week 48 and 96 respectively [13,14].At week 48 the median CD4 increase was 9% (IQR 5-16) and the percentage of patients with HIV-RNA < 400 and < 50 copies/ml were 78 and 64, respectively. At week 96 the median CD4 increased by 14% (IQR:7-19) and the percentage of patients with HIV-RNA < 50 copies/ml was 64.

The dual-PI regimen in our study was chosen due to the genotypic resistance profile and the ongoing virological failure. The highly resistant genotypic profile was mainly due to an extensive exposure to the N(n)RTI class that has been considered the backbone of antiretroviral regimens. The presence of NAMs has been demonstrated to be highly correlated with reduced susceptibility to all N(n)RTIs [19], thus a therapeutic switch was fully justified. Moreover, when this study began, new PIs as tipranavir and darunavir, the new NNRTI etravirine, and entry inhibitors were not yet available. Raltegravir had not been licensed even for adult populations. In addition, this dual boostedPI-based regimen significantly and rapidly decreased HIV-RNA and led to an improved prognosis for the patients as well as preventing a worsening of the genotypic resistance.

The regimen was well tolerated and none of the patients discontinued the therapy due to side effects. Hepatic parameters remained stable overtime apart from TB, as expected. Lipids increased over 36-42 months of observation without causing therapeutic interruptions.

Hyperlipidemia is a well-know complication of ARV therapy in pediatric patients. The European Pediatric Lipodistrophy Group showed that PI use, specifically ritonavir, was associated with dyslipidemia [18]. Hypercholesterolemia showed a prevalence of 38% in children that took ritonavir vs 24% in those without ritonavir use. Recently, Alam et al showed a prevalence of 31% of metabolic abnormalities in a cohort of 385 HIV-infected children: 4 out of 5 of these children had hypertriglyceridemia and 2 out of 5 of these children had hypercholesterolemia. The use of ritonavir-boosted PI regimens was associated with a consistent risk factor for all metabolic abnormality outcomes [20]. Other two studies showed an association of the use of a double-PI regimen consisting of LPV/r and SQV in HIV-infected children with a percentage of patients with a median serum cholesterol and triglyceride significantly increased compared to the baseline value. Similarly, our results showed that almost 57% of children treated with ATV/r + fAVP developed hypercholesterolemia and hypertriglyceridemia. These data may be considered more serious than those observed in the previous studies and this could be related to the type of analysis used. In fact, we did not define dyslipidemia using a single cut-off value but lipid values were categorized according to age and gender reference values.

Our study is somehow limited by the small number of subjects assessed. Since this observation was carried out in a small number of subjects within the out-patients clinic, we did not decide to conduct a case-control study.

Moreover, we did not have the possibility to obtain pK data, thus the relationship between drug exposure and toxicity could not be assessed. We understand that TDM studies are of pivotal importance when using drug regimens which include compounds with a potential interaction, *e.g.* 2 PIs.

In this study, we have proposed a therapeutic strategy that could be considered obsolete and most likely is not being used in most pediatric Infectious Disesase divisions. Nevertheless, in the presence of multiple failures, particularly with N (n)RTIs and NNRTIs, this strategy has overcome the complexity of the resistance pattern. This double-boosted protease regimen with ATV/r and fAVP was effective in suppressing HIV viremia and improving immunological outcome. During the study duration, hypercholesterolmia and hypertriglyceridemia were commonly observed and need close follow-up. The impact on bilirubin level and lipid profile suggests caution in using this regimen in the pediatric population. Thus, this regimen should only be considered as a possible option for a rescue treatment in HIV-infected children and adolescents who failed N(n)RTIs and more so in those with an extensive N(n)RTI resistance.

## Conclusions

The main contribution of this study lies in the clinical efficacy of a regimen composed by ATV/r and fAVP in effectively maintaining the viral suppression together with a good immunological outcome. An increase in lipid levels was demonstrated in the majority of children included in our study although none of them discontinued HAART. In the future, safer therapeutic strategies, with the same effectiveness, will ameliorate the performance status of our patients. In the meantime, these children will be safely followed to that point.

## Misc

Stefano Rusconi, Vania Giacomet, Chiara Mameli contributed equally to this work.

## Competing interest

Authors declared no conflict of interest.

## Authors’ contributions

SR, VG and CM composed the manuscript. FA and OV analized the data. AV, GZ and MG revised the manuscript. All authors read and approved the final manuscript.

## Pre-publication history

The pre-publication history for this paper can be accessed here:

http://www.biomedcentral.com/1471-2334/12/179/prepub
